# Splenic Rupture in Pregnancy Necessitating Antepartum Splenectomy

**DOI:** 10.7759/cureus.112543

**Published:** 2026-07-12

**Authors:** Diana Vazquez Parker, Gloria Fernandes, Danielle DeFoe, Amanda S Russo, Elizabeth Artiles, Catherine Igel, Miltiadis Pitsos

**Affiliations:** 1 Obstetrics and Gynecology, Jamaica Hospital Medical Center, Jamaica, USA; 2 Surgery, Jamaica Hospital Medical Center, Jamaica, USA; 3 Research Education and Innovation, MediSys Health Network, Jamaica, USA; 4 Medicine, Ross University School of Medicine, Bridgetown, BRB; 5 Maternal-Fetal Medicine, Northwell Health System, Norwalk, USA

**Keywords:** antepartum emergency, epstein-barr virus, splenectomy, splenic laceration, splenic rupture

## Abstract

Splenic rupture most commonly results from blunt abdominal trauma or splenomegaly associated with infection. Diagnosing splenic injury during pregnancy is particularly challenging because its clinical signs and symptoms overlap with those of other common causes of acute abdominal pain in pregnancy. We report the case of a previously healthy 25-year-old woman (gravida 6, para 2, abortions 3, living 2) at 28 weeks and 5 days of gestation who presented with the acute onset of left upper quadrant (LUQ) abdominal pain. She had sustained mild direct abdominal trauma, followed by three weeks of progressively worsening left-sided chest pain radiating to the shoulder. Clinical evaluation and imaging revealed hemorrhagic shock and hemoperitoneum secondary to splenic injury. The patient underwent a successful exploratory laparotomy and splenectomy, during which a splenic laceration was identified. Postoperatively, she was diagnosed with infectious mononucleosis and subsequently had an uncomplicated full-term delivery. This case highlights the importance of thorough evaluation of acute abdominal pain during pregnancy and demonstrates that splenectomy can be safely performed during the third trimester when clinically indicated.

## Introduction

The spleen is particularly susceptible to injury because of its highly vascular and fragile structure and may rupture when its capsule or parenchyma is disrupted [1,2]. Because the spleen sequesters approximately 25% of the body's platelets and a substantial proportion of red blood cells, splenic rupture constitutes a surgical emergency with a significant risk of life-threatening hemorrhage [1,2]. Patients with splenic rupture typically present with left upper quadrant (LUQ) abdominal pain, referred pain to the left shoulder, hypotension, and signs of hemorrhagic shock. Physical examination commonly reveals abdominal tenderness and distension [2]. The diagnosis is generally established using contrast-enhanced computed tomography (CT), although bedside focused assessment with sonography for trauma (FAST) can facilitate the rapid detection of hemoperitoneum and assist in the initial evaluation [2]. The diagnosis of splenic rupture during pregnancy is particularly challenging because several obstetric and non-obstetric conditions may present with acute abdominal pain and hemorrhagic shock [2,3]. Management may also be complex, as severe splenic injuries often require urgent surgical intervention [2]. Because splenic rupture during pregnancy is exceedingly rare, its true incidence and associated maternal and fetal mortality remain unknown. Nevertheless, delayed recognition may result in catastrophic outcomes for both the mother and fetus.

Although most splenic ruptures result from blunt abdominal trauma, splenomegaly associated with Epstein-Barr virus (EBV) infection causing infectious mononucleosis is also a recognized predisposing factor [2,4]. Previous reports have described splenic rupture during pregnancy following trauma [5-8], in association with autoimmune hematologic disorders [9,10], structural splenic abnormalities [11,12], parasitic disease [6], or in the absence of an identifiable precipitating factor [6]. We present a case of splenic rupture in which pregnancy, acute EBV infection, and mild blunt abdominal trauma may each have contributed to the patient's presentation, ultimately necessitating antepartum splenectomy.

## Case presentation

**Figure 1:**
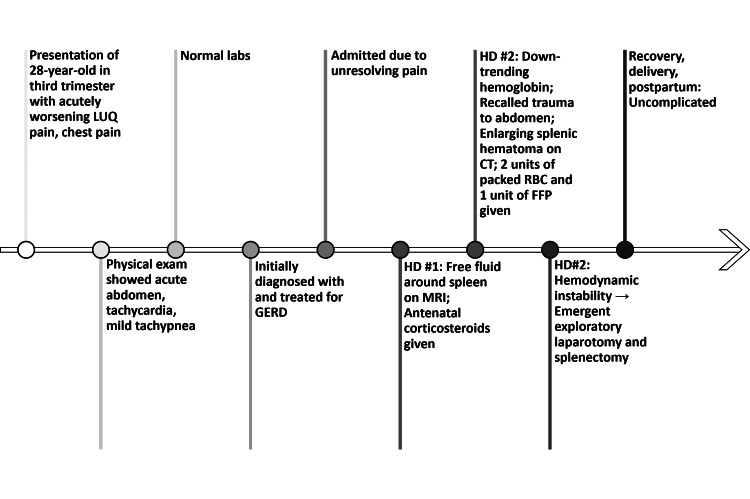
Timeline of case presentation CT: computed tomography, FFP: fresh frozen plasma, GERD: gastroesophageal reflux disorder, HD: hospital day, MRI: magnetic resonance imaging, RBC: red blood cells.

Written informed consent was obtained from the patient. A timeline of clinical events is provided in Figure 1. A 25-year-old woman (gravida 6, para 2, abortions 3, living 2) at 28 weeks and 5 days of gestation presented with acutely worsening LUQ pain, left-sided chest pain radiating to the shoulder that had been present for three weeks, constipation, and nausea. She initially denied any history of abdominal trauma. She resided in a shelter and had a past medical history of an untreated *Klebsiella* urinary tract infection (UTI), intermittent asthma controlled with albuterol, and two prior deliveries: one vaginal delivery, followed by one cesarean section. On presentation, she was tachypneic (22 breaths/min) and tachycardic (122 beats/min), with otherwise normal vital signs. She appeared pale, diaphoretic, and in acute distress. Abdominal examination revealed a distended, tympanic abdomen without rebound tenderness. Fetal assessment demonstrated a reactive nonstress test with irregular uterine contractions, and the cervical os was closed. Initial laboratory evaluation showed no significant leukocytosis (white blood cell count, 14 × 10³/µL) or electrolyte abnormalities. Despite her pale appearance on physical examination, her initial hemoglobin level was 10.8 g/dL. Cardiac evaluation was unremarkable. Gastroesophageal reflux disease (GERD) was initially considered the most likely diagnosis, and she was treated with intravenous famotidine (20 mg), morphine as needed for pain control, and oral aluminum hydroxide-magnesium hydroxide with simethicone (30 mL).

**Figure 2:**
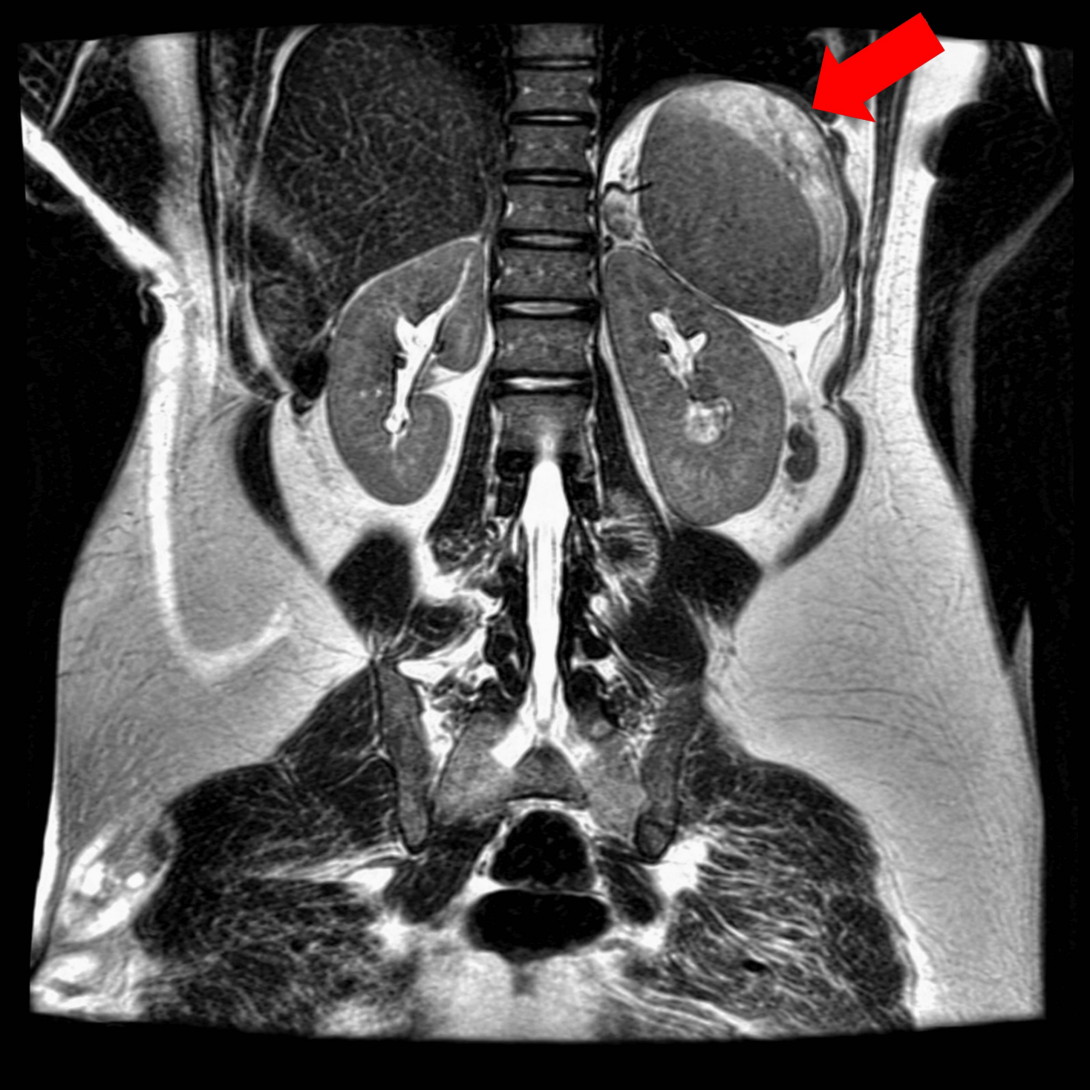
MRI of the abdomen and pelvis showing splenic hemorrhage The spleen measures 10.2 cm in length. The red arrow points to the perisplenic and subdiaphragmatic fluid collection, which demonstrates intermediate signal intensity on T2-weighted images.

She was admitted because of persistent, unresolved pain. A bedside FAST examination was interpreted as negative, although the study was limited by the gravid uterus and the patient's pain. Chest radiography showed no evidence of free intraperitoneal air. Abdominal ultrasonography demonstrated hepatomegaly and ascites. Magnetic resonance imaging (MRI) of the abdomen and pelvis without contrast revealed free fluid surrounding the spleen, raising suspicion for perisplenic hemorrhage; however, the splenic artery was not visualized (Figure 2). Consequently, the examination was considered inconclusive for the evaluation of a possible splenic artery aneurysm. Because of the possibility of preterm delivery, the patient received a two-dose course of intramuscular betamethasone (12 mg per dose). Her urinary tract infection was treated with a single 2-g dose of intravenous cefazolin, followed by nitrofurantoin (Macrobid) 100 mg orally for seven days.

**Figure 3:**
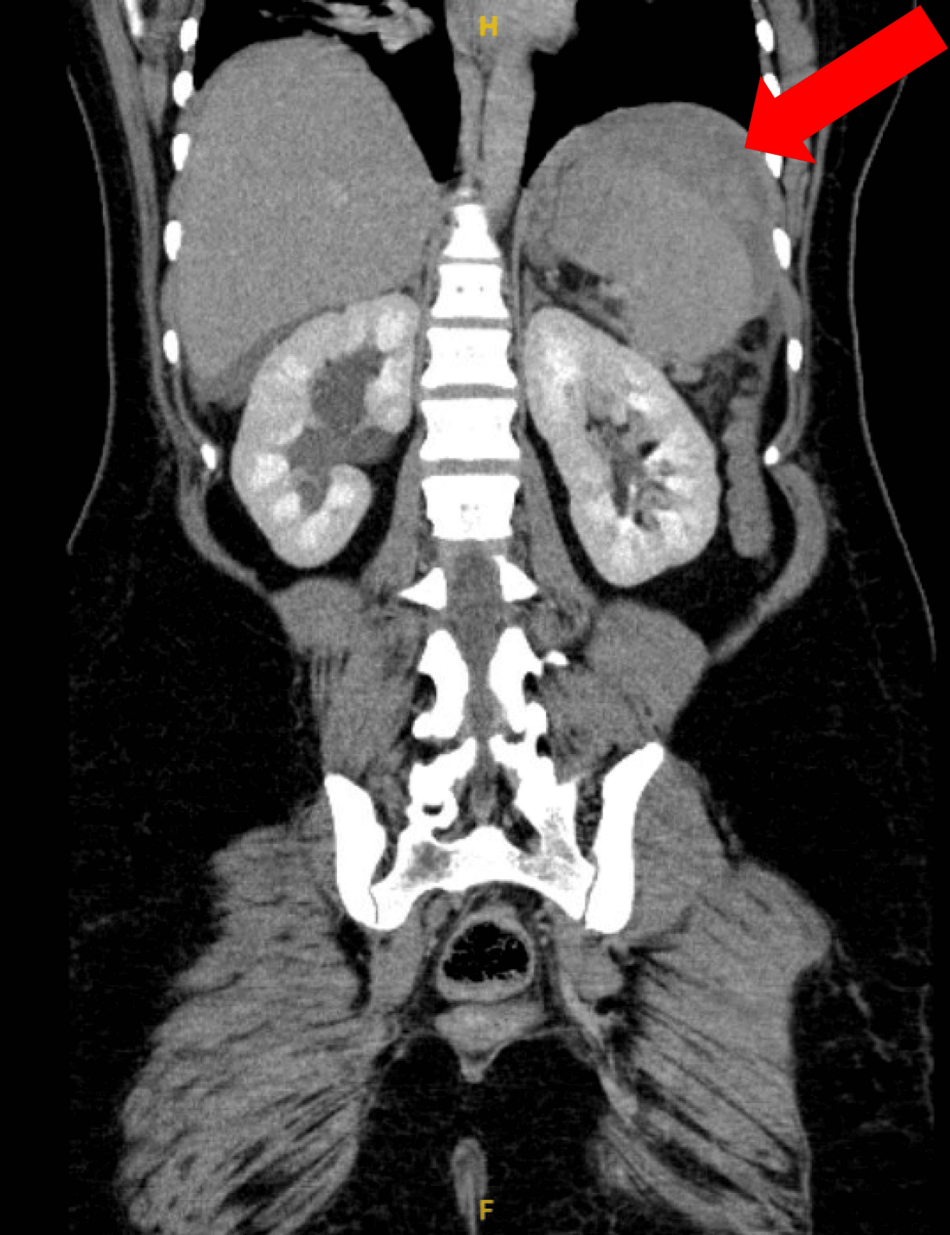
Contrast-enhanced CT of the abdomen and pelvis demonstrating a large perisplenic hematoma (coronal view) The red arrow indicates irregularity of the inferior aspect of the spleen and a large amount of perisplenic hypodense fluid, consistent with hemoperitoneum.

The following day, abdominal examination revealed marked rebound tenderness. Bedside ultrasonography demonstrated a normal-appearing placenta without evidence of a retroplacental hematoma. Fetal heart rate tracing remained reactive, with no decelerations. An emergent contrast-enhanced CT scan of the abdomen and pelvis demonstrated a large perisplenic hematoma, an irregular contour of the inferior aspect of the spleen, and hemoperitoneum involving Morrison's pouch, without evidence of a retroplacental hematoma (Figure 3). At that time, the patient recalled that, before the onset of her symptoms, her six-year-old son had jumped onto the left side of her abdomen while playing. Her hemoglobin level had decreased to 7.7 g/dL, and she was treated with a transfusion of two units of packed red blood cells and one unit of fresh frozen plasma.

**Figure 4:**
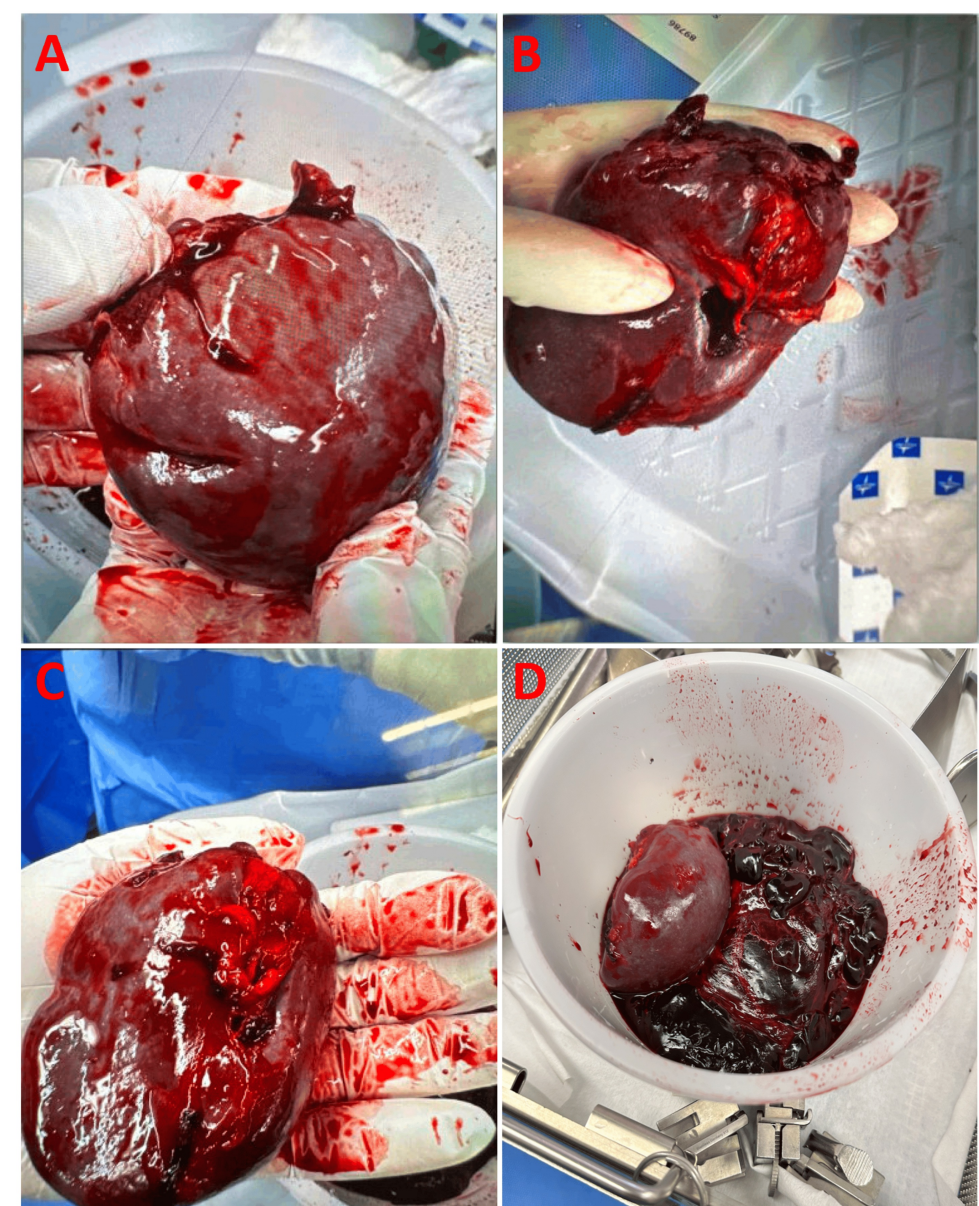
Intraoperative images: (A) anterior aspect of the spleen, (B, C) posterior aspect of the spleen, and (D) spleen with evacuated blood clots

Given the imaging findings, hemodynamic instability, and the need to prioritize maternal resuscitation and hemorrhage control, the patient underwent an emergent exploratory laparotomy with splenectomy under general anesthesia. Obstetricians and neonatologists were present throughout the procedure to provide continuous fetal monitoring and to perform an emergency cesarean delivery if clinically indicated. Intraoperatively, hemoperitoneum with a large perisplenic hematoma and overlying blood clots was identified (Figure 4). The procedure was completed without complications. The patient was transferred to the post-anesthesia care unit and subsequently to the surgical intensive care unit (SICU) in stable condition. A 19-French Blake drain was left in the LUQ, and continuous fetal monitoring was maintained using an external Doppler transducer and tocodynamometer.

Following surgery, an EBV serologic panel was obtained and was positive for EBV viral capsid antigen immunoglobulin G (IgG) antibody (418 U/mL) and EBV nuclear antigen antibody (600 U/mL). In addition, the Monospot test was positive, suggesting that infectious mononucleosis may have contributed to the splenic rupture. Gross pathological examination showed that the spleen weighed 123.6 g and measured 8 × 6 × 4 cm. The splenic capsule was gray and smooth, with a linear tan, shaggy area of capsular disruption measuring 3 × 0.5 cm on the posterior aspect of the visceral surface. Separate blood clots measuring 16 × 12 × 4 cm and weighing 366.7 g were also identified.

The patient remained in the SICU, where she received prophylactic intravenous magnesium sulfate for tocolysis through postoperative day 1 (POD1). Nonstress testing was performed three times daily. On POD3, she was transferred to the antepartum unit. Her postoperative ileus resolved by POD4, and she was cleared for discharge from a surgical standpoint. However, a routine urine culture grew extended-spectrum β-lactamase-producing *Klebsiella*, necessitating intravenous ertapenem (1 g every 24 hours) for 14 days; therefore, she remained hospitalized to complete treatment. She was advised to receive routine post-splenectomy vaccinations and was discharged on POD14.

The patient recovered well postoperatively and was followed weekly in a high-risk obstetric clinic. A repeat urine culture obtained at 33 weeks and 4 days of gestation demonstrated persistent *Klebsiella bacteriuria*, and she was readmitted for a 21-day course of intravenous ertapenem (1 g daily). The remainder of the pregnancy was uncomplicated. At 39 weeks and 2 days of gestation, she underwent an elective repeat cesarean delivery with bilateral tubal sterilization. Her postpartum course was uncomplicated, and she was discharged on POD3.

## Discussion

Despite the rarity of splenic laceration in the absence of previously diagnosed splenic disease or major trauma, clinicians should maintain a high index of suspicion in patients presenting with hypovolemic shock, LUQ tenderness, generalized peritonitis, and/or referred pain to the left shoulder [2,13]. Pregnancy further complicates the diagnostic evaluation because several obstetric emergencies, including uterine rupture and placental abruption, may present with similar features [2,3]. In a review of 607 cases of splenic rupture, approximately 6% were pregnancy-related and 4% were trauma-related [13]. Among the pregnancy-associated cases, most occurred without documented trauma, whereas all trauma-related cases involved only minor traumatic events [13]. These findings underscore the importance of considering splenic injury in any pregnant patient presenting with acute abdominal pain, particularly when accompanied by signs of hemorrhage or hemodynamic instability.

Our patient presented with the unusual combination of pregnancy, possible EBV-associated splenic vulnerability, and minor blunt abdominal trauma, emphasizing that multiple contributing factors may coexist. Although the remote traumatic event was not initially disclosed, its potential contribution to the splenic injury should not be underestimated. Furthermore, despite the absence of splenomegaly on pathological examination, EBV infection may have lowered the threshold for splenic rupture in the setting of pregnancy and minor trauma. This case therefore illustrates that splenic rupture may result from the interaction of multiple predisposing factors rather than a single identifiable cause and highlights the importance of maintaining clinical suspicion even when the etiology is not immediately apparent.

Atraumatic splenic rupture has been reported in association with several pathological conditions in addition to EBV infection, including malaria, particularly in endemic regions [14,15]. Splenic enlargement and vascular congestion associated with malaria may predispose the spleen to rupture. Hematologic malignancies, including leukemia and lymphoma, have also been implicated through malignant infiltration and disruption of normal splenic architecture [16-18]. Although neither malaria nor hematologic malignancy was present in our patient, awareness of these conditions is important when evaluating pregnant patients with acute abdominal pain, hemoperitoneum, or unexplained hemorrhagic shock.

Several reports have described splenectomy for traumatic splenic injury during pregnancy [7,8]. However, those cases involved patients who sustained significant acute trauma, in contrast to our patient, whose history was notable only for remote minor blunt abdominal trauma that was recalled after further questioning. For example, one report described a successful laparoscopic splenectomy in a 25-year-old woman at 18 weeks of gestation following a high-speed motor vehicle collision, with an uncomplicated recovery and subsequent antenatal course [7]. Another report described a patient who was struck by a commercial bus at 23 weeks of gestation and underwent open splenectomy for splenic rupture, with favorable maternal and fetal outcomes [8]. In contrast, the absence of an initially recognized traumatic event in our patient made the diagnosis considerably more challenging. Within the context of the existing literature, this case highlights that seemingly trivial or remote trauma may contribute to splenic injury and should be explored carefully during the clinical evaluation.

Although splenectomy during pregnancy has traditionally been considered a high-risk procedure, a growing body of evidence suggests that it can be performed safely when clinically indicated [9-12]. Previously reported cases have involved indications other than traumatic splenic rupture, including medication-refractory immune thrombocytopenic purpura (ITP) [9,10], wandering spleen [11], and large splenic cysts [12], with favorable maternal, fetal, and postpartum outcomes. Collectively, these reports, together with the present case, support the feasibility of antepartum splenectomy when prompt surgical intervention is necessary. Nevertheless, successful conservative management has also been reported in selected patients [19,20]. Therefore, management should be individualized, with treatment decisions guided by maternal hemodynamic status, fetal condition, the severity of splenic injury, and the overall clinical scenario.

## Conclusions

This case illustrates the diagnostic challenges of identifying splenic laceration and the management of splenic injury requiring splenectomy during pregnancy. Initial conservative diagnostic and therapeutic approaches were pursued for acute abdominal pain, including treatment for presumed GERD, despite the patient's significant clinical distress. However, persistent symptoms and inconclusive MRI findings ultimately necessitated a CT, which established the diagnosis of splenic laceration. This case adds to the growing body of evidence suggesting that splenectomy can be performed safely during pregnancy when clinically indicated, as demonstrated by this patient's successful progression to a full-term delivery following surgery.
